# Color matching and metameric behavior of composite resins applied on anodized titanium surfaces: an in vitro spectrophotometric study

**DOI:** 10.1186/s12903-026-08118-8

**Published:** 2026-03-18

**Authors:** Umur Davut, Ozge Doganay-Ozyilmaz, Ozgun Yusuf Ozyilmaz

**Affiliations:** 1 Department of Prosthodontics, Institute of Health Sciences, Bezmialem Vakıf University, Adnan Menderes Bulvarı Vatan Caddesi, Istanbul, Fatih 34093 Turkey; 2 Department of Oral and Maxillofacial Surgery, Faculty of Dentistry, Bezmialem Vakıf University, Istanbul, Turkey; 3 Department of Prosthodontics, Faculty of Dentistry, Istanbul Galata University, Istanbul, Turkey

**Keywords:** Titanium anodization, Composite resin, Color matching, Metamerism, Dental implant, CIEDE2000

## Abstract

**Background:**

The aim of this study was to evaluate the color matching and metameric behavior of two different composite resins applied to titanium surfaces anodized to yellow and pink colors. The study aimed to determine the effects of anodization color and composite resin type on color performance in hybrid prosthesis and screw access hole closure applications.

**Materials and methods:**

A total of 72 Grade 5 titanium discs (10 mm diameter × 2 mm thickness) were allocated to three anodization conditions: nonanodized/gray (0 V, *n* = 24), yellow (55 V, *n* = 24), and pink (80 V, *n* = 24). Each anodization group was subdivided into two subgroups: 3 M Filtek Z250 (microhybrid) and Tokuyama Estelite Sigma Quick (supra-nano hybrid) composite resins (A2 shade, 2 mm thickness; *n* = 12 per subgroup). Gray titanium + composite specimens were used as the reference for ΔE₀₀ calculations; yellow and pink anodization groups constituted the experimental groups. Color measurements were performed using a spectrophotometer (Konica Minolta CM-3700 A) under four light sources (D65, D50, incandescent A, and F11). Data were analyzed using mixed-design repeated-measures ANOVA, Bonferroni post hoc test, and independent samples t-test (α = 0.05).

**Results:**

Anodization color (η² = 0.941) and composite type (η² = 0.952) demonstrated strong effects on ΔE₀₀ values (both *p* < 0.001). Light source showed statistically significant but clinically negligible effect (η² = 0.521, mean difference < 0.1 ΔE₀₀). Pink anodization exhibited higher color differences than yellow (2.84–2.99 vs. 1.53–1.62), and Tokuyama composite higher than 3 M (2.89–3.12 vs. 1.41–1.54) (both *p* < 0.001). Yellow+3 M combination showed lowest values (0.91–0.97), while Pink+Tokuyama showed highest (3.68–3.97). Metamerism index remained minimal (MI < 0.21).

**Conclusions:**

Careful material selection is essential for optimal esthetics in anodized titanium-composite systems. Yellow anodization combined with a microhybrid composite demonstrated the most favorable color matching performance under the tested conditions. for hybrid prostheses, screw-retained restorations, and esthetic implant applications.

**Trial registration:**

Not applicable.

## Introduction

Titanium and titanium alloys are among the most widely used materials in biomedical applications owing to their superior biocompatibility, high corrosion resistance, and mechanical strength [1]. The long-term success of high-purity Grade 5 titanium (Ti-6Al-4 V) has been demonstrated in numerous studies, particularly in dental implants, orthopedic prostheses, and surgical instruments [2, 3]. However, the natural gray tone of titanium may prove inadequate in applications where high esthetic demands are expected and may negatively affect patient satisfaction [4].

To overcome this problem, the anodization process was developed, which forms a controlled oxide layer on the Grade 5 titanium surface, enabling the material to exhibit various colors [5]. Because the coloration achieved by anodization occurs through an optical interference mechanism without the use of pigments or dyes, it provides a biocompatible and long-lasting solution [6]. Furthermore, the ability to precisely control the oxide layer thickness during anodization allows specific colors to be produced in a reproducible manner [7]. In dental implant applications, yellow anodization is used to mimic dentin color, whereas pink anodization is preferred for achieving esthetic harmony with peri-implant soft tissues. These two colors are included in the standard anodization palettes of major implant manufacturers such as Nobel Biocare and Straumann and are widely used worldwide [8].

Nevertheless, it is well established that esthetic success is related not only to the appearance of the surface color under a single type of illumination but also directly to the color stability of the material under various lighting conditions [9]. Metamerism describes the phenomenon in which the perceived color of a material varies under different light sources and constitutes a critical problem, particularly in esthetic restorations [10]. Metameric differences may cause patients to perceive color variations in their restorations under natural light, fluorescent illumination, or artificial light conditions [11]. This may adversely affect both patient satisfaction and long-term esthetic outcomes [12].

The titanium–composite resin combination in implant-supported restorations is routinely used in three main clinical scenarios. First, in implant-supported bar-retained hybrid prostheses, artificial teeth and gingiva made of composite resin are applied onto a titanium bar substructure [13]. This approach provides significant weight and cost advantages over all-ceramic restorations. Second, in screw-retained implant crowns, screw access channels are sealed with composite resin [14]. The depth of these channels typically ranges between 2 and 3 mm and sealing with composite is necessary for both functional and esthetic reasons. Third, chip fractures occurring in monolithic zirconia or metal–ceramic restorations over anodized titanium bars are restored via intraoral composite repair [15, 16].

Composite resin layer thickness is critically important for masking the substructure color and ensuring color stability [17, 18]. A minimum composite thickness of 2 mm is recommended to achieve adequate optical opacity over a metal substrate [19]. This thickness represents both the clinical standard for posterior composite restorations and an appropriate depth for screw access hole sealing procedures [20]. The selection of a 2 mm thickness in the present study was based on these clinical standards and literature findings.

The addition of composite materials onto anodized surfaces further complicates the overall optical characteristics of the system [21]. The matrix structure, filler particles, and light absorption–reflection properties of composites may interact with the anodized color of titanium, potentially producing unexpected metameric effects [22]. Therefore, evaluating the color stability of different composite materials applied after anodization under various light sources is of great importance for both material selection and accurate clinical decision-making [23].

Particularly in individuals with a thin gingival phenotype, the distinct gingival morphology in peri-implant regions facilitates reflection of the metallic substructure, making color discrepancies more noticeable to patients [4]. In implant-supported fixed prostheses, the optical properties of the composite resin used to seal the screw access hole and the color of the underlying titanium surface directly influence the esthetic outcome, further highlighting the importance of research in this area [24]. Evaluating composite materials applied to titanium surfaces modified with different anodization colors in terms of light-dependent color differences and metameric behavior, especially in esthetic regions, is important for enhancing clinical success and ensuring patient satisfaction [25].

The aim of this study was to evaluate the color matching and metameric behavior of two different composite resins (3 M Filtek Z250 and Tokuyama Estelite Sigma Quick; A2 shade) applied onto Grade 5 titanium surfaces anodized to pink and yellow colors under four different standard light sources (D65, D50, F11, and incandescent). The study aimed to provide directly applicable information for hybrid prosthesis, screw access hole closure, and composite repair applications on anodized titanium surfaces, which are frequently encountered in daily clinical practice.

### Study hypotheses

The null hypotheses of this study were as follows:

1. Anodization color (yellow vs. pink) does not affect ΔE₀₀ values when composite is applied onto the surface.
2. Composite type (3M Filtek Z250 vs. Tokuyama Estelite Sigma Quick) does not affect ΔE₀₀ values on anodized titanium.
3. Light source variation (D65, D50, incandescent, F11) does not affect the ΔE₀₀ values of titanium–composite combinations.
4. Under different light sources, titanium–composite combinations exhibit clinically acceptable metameric behavior (metamerism index < 0.8).

## Materials and methods

### Study design

In this in vitro study, color matching and metamerism were evaluated in specimens prepared by applying different composite resin materials onto Grade 5 titanium discs anodized to yellow (55 V) and pink (80 V) colors. Specimens prepared by applying the same composite materials onto nonanodized (gray, 0 V) titanium discs were used as the reference standard for ΔE₀₀ calculations. The study was conducted at the laboratory of the Department of Prosthodontics, Faculty of Dentistry, Bezmialem Vakif University.

### Reference group selection

Specimens prepared by applying composite onto nonanodized (gray, 0 V) titanium discs were used as the reference for all ΔE₀₀ calculations. This selection was intended to isolate the optical effect of anodization. Had natural tooth or gingival color been used as the reference, the resulting color difference would have reflected the combined effects of both composite translucency and anodization, making it impossible to determine the specific contribution of anodization.

Gray titanium provides a standardized baseline owing to its spectrally neutral and consistent reflectance properties. This methodology is consistent with previous studies investigating the optical behavior of anodized titanium [6, 21]. Furthermore, in clinical procedures such as screw access channel closure in implant-supported prostheses, intraoral composite repair in monolithic ceramic restorations, and composite application in hybrid prostheses, the titanium substructure may be used in either anodized or nonanodized form during the fabrication stage, and the present study evaluates the effect of this choice on composite color performance.

### Composite resin and shade selection

Two different composite resin materials were selected:

- 3M Filtek Z250 (3M ESPE, St. Paul, MN, USA): Microhybrid composite, mean particle size 0.6 µm, A2 shade [26]
- Tokuyama Estelite Sigma Quick (Tokuyama Dental, Tokyo, Japan): Supra-nano hybrid composite, mean particle size 0.2 µm (spherical filler), A2 shade [22]

#### Composite type selection

This selection was made to evaluate the effect of different filler morphologies (microhybrid vs. supra-nano) on titanium substrate color reflection and to represent commonly used composite types in clinical practice.

#### A2 shade selection

Both composites were used in A2 shade. This selection was based on the following rationale:

1. A2 is one of the most common natural tooth shades in the VITA Classical shade guide and is frequently preferred in implant-supported restorations. This prevalence ensures the applicability of the findings to a broad patient population [27].
2. As a mid-value shade, it is ideal for evaluating the optical effect of the titanium substrate. In excessively light shades (e.g., A1, B1), the substrate effect may be masked, whereas in excessively dark shades (e.g., A3.5, C4), the substrate color contribution may be exaggerated or overlooked [28].
3. It is widely used as a standard control shade in composite color research, ensuring the comparability of the findings with the literature [29, 30].

### Composite thickness rationale

The selection of a 2 mm composite thickness was based on the following clinical and scientific rationale:

1. The recommended minimum clinical standard thickness for posterior composite restorations [31].
2. The minimum thickness providing adequate optical opacity for metal substrate masking [32].
3. The typical depth of screw access channels in screw-retained implant restorations (2–3 mm) [33].

### Anodization voltage rationale

The anodization voltages of 55 V (yellow) and 80 V (pink) were selected on the basis of their compatibility with tissue color matching and commonly used anodization parameters in clinical applications [6, 8].

### Light source selection

Four different light sources were used. These illuminants represent the main light sources defined in the ISO 3664:2009 standard and cover the lighting conditions encountered in daily life [12, 34]:

- D65 (6500 K): Cool daylight (reference illuminant).
- D50 (5000 K): Neutral daylight.
- Incandescent A (2856 K): Incandescent lamp, home environment.
- F11 (4000 K): Fluorescent lamp, office/clinical environment.

### Sample size calculation

Sample size calculation was performed using G*Power 3.1.9.7 software (Heinrich-Heine-Universität Düsseldorf, Germany) [35]. Using an effect size of f = 0.333, alpha α = 0.05, and power (1 – β) = 0.95, the minimum sample size was determined as *n* = 12 per experimental group.

### Specimen preparation and grouping

#### Titanium disc fabrication

**Fig. 1:**
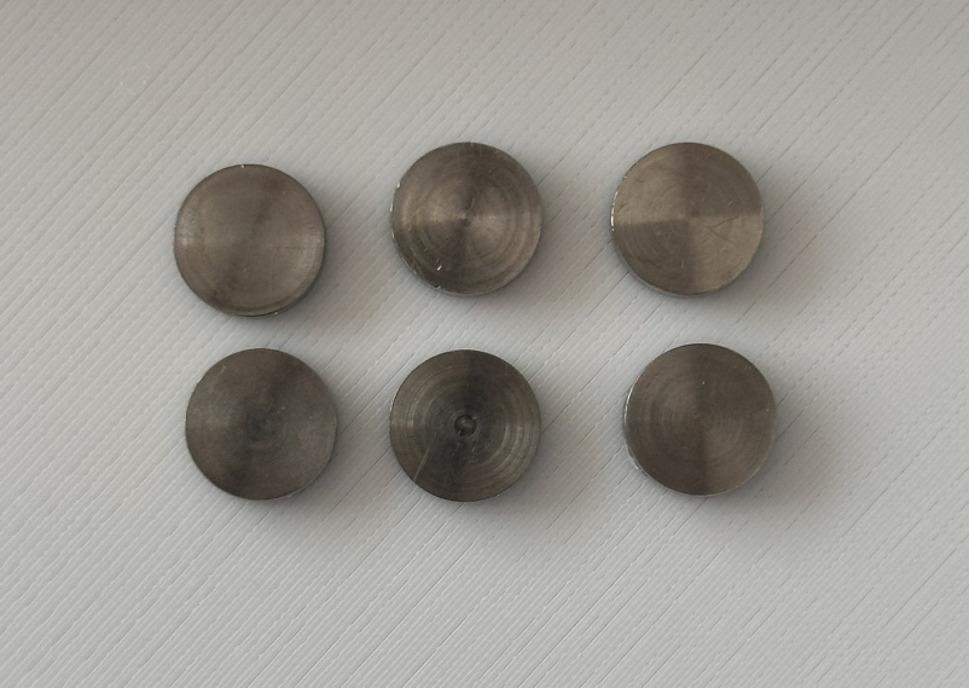
Representative image of non-anodized (gray) Grade 5 titanium reference discs used in the study

A total of 72 discs, 10 mm in diameter and 2 mm in thickness, were fabricated from Grade 5 titanium alloy (Ti-6Al-4 V; ASTM B348 Grade 5; Alfa Aesar, Haverhill, MA, USA) via precision CNC turning (Fig. 1). All discs were cut from the same titanium bar stock and pre-polished with 800-, 1000-, and 1200-grit silicon carbide papers to ensure surface homogeneity. Disc thicknesses were verified using a digital caliper to ensure a tolerance of 2.0 ± 0.1 mm.

### Grouping Scheme

The 72 discs were grouped as follows:

#### Group 1 – Reference (Gray titanium, 0 V)

24 discs – 12 discs + 3 M Filtek Z250 composite; 12 discs + Tokuyama Estelite Sigma Quick composite. *This group was used solely as the reference for ΔE₀₀ calculations and was not included in statistical comparisons.*

#### Group 2 – Yellow anodized (55 V)

24 discs – 12 discs + 3 M Filtek Z250 composite; 12 discs + Tokuyama Estelite Sigma Quick composite.

#### Group 3 – Pink anodized (80 V)

24 discs – 12 discs + 3 M Filtek Z250 composite; 12 discs + Tokuyama Estelite Sigma Quick composite.

#### TOTAL

6 combinations × 12 discs = 72 discs.

#### Statistical analysis

4 experimental groups × 12 discs = 48 discs (gray reference excluded).

### Randomization procedure

Before allocation to each anodization condition, the titanium discs were numbered from 1 to 72 and assigned to three anodization groups (gray, yellow, and pink) using a random number generator (Microsoft Excel 2019, RAND function). Within each anodization group, the discs were re-randomized and allocated to two composite subgroups.

The operator performing color measurements was not blinded to the experimental group assignment because the anodization color was visually distinguishable. However, to ensure objectivity during measurements, specimens were coded and the spectrophotometer recorded values automatically.

### Surface preparation and anodization

#### Surface cleaning

Each disc was subjected to a three-stage cleaning protocol to remove surface particles and grease residues:

1. Acetone bath (10 min): To remove organic residues from the surface, the discs were placed in an ultrasonic cleaner (Branson 2800, Branson Ultrasonics, Danbury, CT, USA) in acetone for 10 min.
2. Isopropyl alcohol bath (10 min): A second ultrasonic cleaning step was applied to remove grease and solvent residues.
3. Distilled water bath (10 min): In the final cleaning step, the discs were rinsed in pure distilled water and dried at room temperature.

### Anodization procedure

For preparation of the electrolytic solution, 2 g of trisodium phosphate (Na₃PO₄; Akbel Kimya, Turkey) was weighed on a precision balance (Weather Forecast-THR124, China) and dissolved in 500 ml of distilled water (UPS Ilac, Turkey) [5].

Anodization was performed using a Smartgift 120 V adjustable DC power supply (Smartgift, China). The system was set up as follows: the titanium disc was connected to the anode terminal, and a stainless-steel plate (20 mm × 20 mm, AISI 304) was connected to the cathode terminal. The two terminals were placed 30 mm apart within the electrolyte without contact. Room temperature (23 ± 2 °C) was maintained throughout the process, and the electrolyte was continuously homogenized using a magnetic stirrer.

#### Anodization parameters [5]

**Fig. 2:**
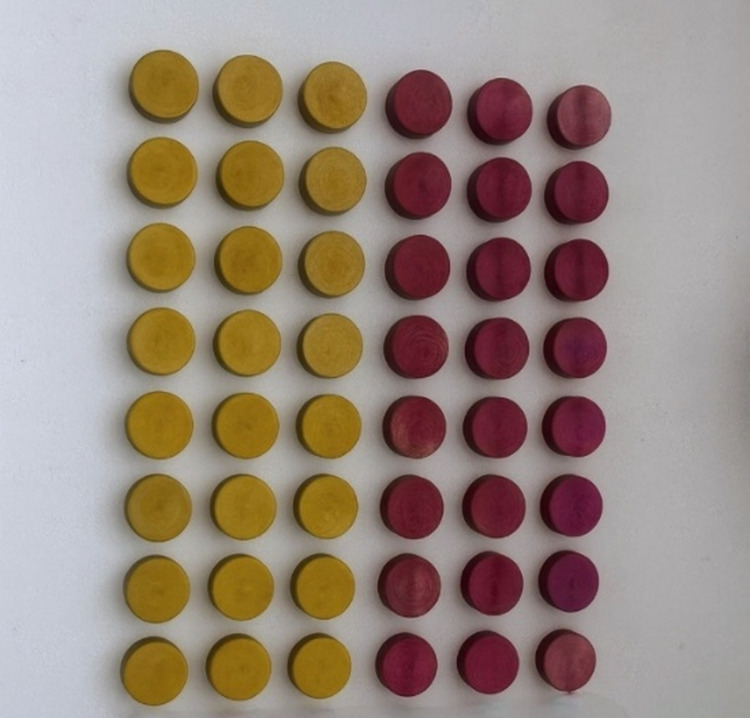
Representative images of anodized titanium specimens showing yellow (55 V, left panel) and pink (80 V, right panel) anodization colors achieved through electrochemical oxidation

Gray group: No anodization applied (0 V); Yellow group: 55 V constant voltage, 1 min; Pink group: 80 V constant voltage, 1 min (Fig. 2).

After anodization, the specimens were rinsed thoroughly with distilled water (3 × 100 ml) to remove electrolyte residues and dried in the dark at room temperature for 24 h. All prepared titanium discs were stored in numbered containers in a moisture-free dark environment until composite resin application.

### Composite resin application

#### Bonding procedure

A thin layer of Bisco Metal Primer (Bisco Inc., Schaumburg, IL, USA), specifically developed for metal surfaces, was applied to each disc surface using a bonding brush and left at room temperature for 60 s to allow complete solvent evaporation.

### Composite application

Each composite material was placed onto the disc surface in a single layer using a custom-made silicone mold (10 mm diameter × 2 mm thickness). The mold was used to standardize both the thickness and surface smoothness of the specimens. To ensure a homogeneous flat layer and eliminate air bubbles, a transparent glass plate (microscope coverslip) was placed on top of the silicone mold and light pressure was applied.

### Polymerization procedure

Composite resins were polymerized using a DTE O-light LED curing unit (Woodpecker, Guilin, China) according to the manufacturer’s instructions. Device specifications: wavelength 385–515 nm; light intensity 1000–1200 mW/cm² (standard mode); tip diameter 8 mm.

Light intensity was verified before each polymerization session using a digital radiometer (Bluephase Meter II, Ivoclar Vivadent, Schaan, Liechtenstein), and all measurements were confirmed to be within the range of 1050 ± 50 mW/cm².

Polymerization protocol: The curing unit tip was held perpendicular to the composite surface at a distance of 2 mm, and light curing was performed for 20 s. The distance was standardized using a custom-made 2 mm thick spacer. The same operator performed all polymerization procedures to ensure reproducibility.

Each specimen was stored in distilled water at 37 °C in the dark for 24 h after polymerization. This procedure was performed to allow the composites to reach a stable color equilibrium following water sorption and to facilitate diffusion of residual monomers.

### Finishing and polishing

For finishing and polishing, specimen surfaces were ground using an automatic grinding machine (Minitech 233, PRESI GmbH, Germany) under water cooling at 100 rpm with silicon carbide papers of 600, 800, 1000, 1200, and 1500 grit sequentially, for 10 s per grit.

Specimen thicknesses were verified by measuring at five different points using a digital caliper (Mitutoyo, Tokyo, Japan; accuracy ± 0.01 mm). Through this process, the 0.2 ± 0.1 mm excess composite obtained with the silicone mold was ground away, achieving a final composite thickness of 2.0 ± 0.05 mm.

After polishing, the specimens were placed in an ultrasonic bath with distilled water for 10 min for surface cleaning and then dried with oil-free air. All specimens were stored in the dark at room temperature for 24 h before color measurement.

### Color measurement and analysis

#### Spectrophotometer setup

Color measurements were performed using a Konica Minolta CM-3700 A spectrophotometer (d/8° geometry, SCI mode, 10° observer, 8 mm MAV aperture, 360–740 nm/10 nm interval) [36]. The device was calibrated before each measurement session using white and black calibration tiles under the D65 illuminant standard in accordance with the manufacturer’s instructions.

### Measurement standardization

**Fig. 3:**
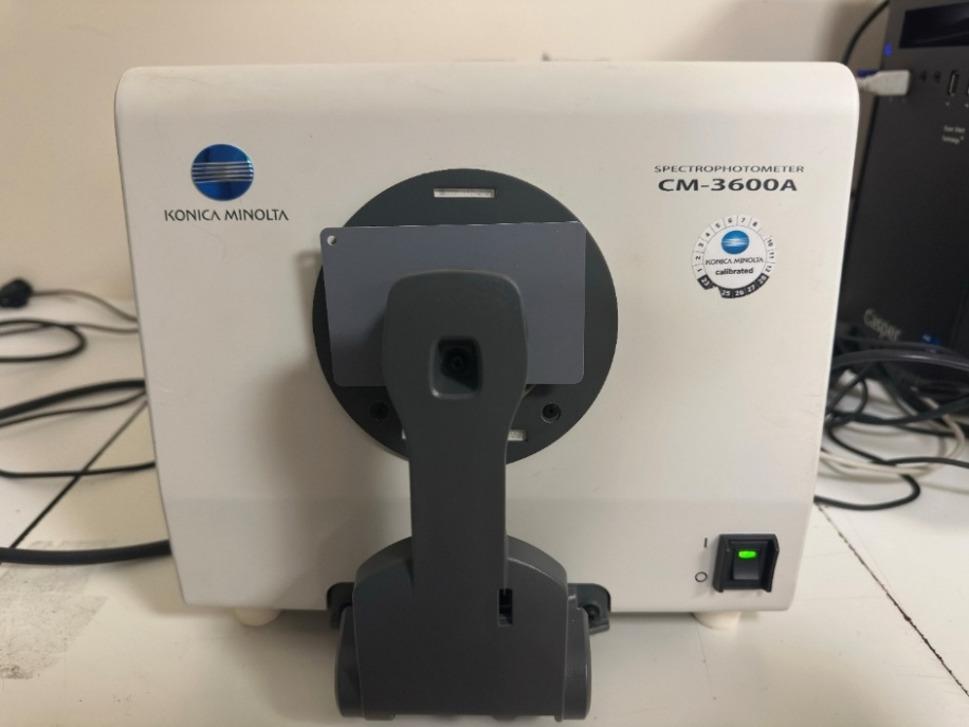
Spectrophotometric color measurement setup demonstrating standardized specimen positioning against an 18% gray card background

During measurements, a neutral gray card (18% reflectance; Gray Card) was placed behind each specimen to eliminate background effects and ensure optical standardization [37]. This method was used to minimize color measurement errors that could arise from the semi-translucent nature of composite materials (Fig. 3).

Three replicate measurements were taken at the same position for each specimen. To improve measurement repeatability, specimen surfaces were cleaned with oil-free air before measurement, and a 30-second rest interval was allowed between each measurement.

All measurements were performed in a light-proof black measurement room, completely isolated from ambient light.

### Multiple illuminant measurements

Measurements for each specimen were taken under four different standard illuminants: D65 (6500 K): Cool daylight; D50 (5000 K): Neutral daylight; Incandescent A (2856 K): Incandescent lamp, home environment; F11 (TL84) (4000 K): Fluorescent lamp, office/clinical environment.

#### Important note

The spectrophotometer measured the spectral reflectance data (360–740 nm) of each specimen, and the device software calculated CIE L*a*b* values for each illuminant using these spectral data. The physical light source was not changed; all calculations were derived from the spectral reflectance curve (standard colorimetric practice). The term “D65 illuminant standard” in the calibration description refers to the initial reference calibration of the device.

#### Color difference calculation

Using the obtained L*, a*, b* values, the color difference of each specimen relative to the reference gray titanium disc was calculated according to the CIEDE2000 color difference (ΔE₀₀) formula [12]:

$$\triangle{\mathrm E}_{\circ\circ}\;=\;\surd\lbrack(\triangle\mathrm L'/\mathrm{klSl})^2\;+\;(\triangle\mathrm C'/\mathrm{kcSc})^2\;+\;(\triangle\mathrm H'/\mathrm{khSh})^2\;+\;\mathrm{RT}(\triangle\mathrm C'/\mathrm{kcSc})(\triangle\mathrm H'/\mathrm{khSh})\rbrack$$

Here, kl, kc, and kh are parametric factors used as correction terms to adjust viewing condition values. In this study, the parametric factor values were set to 1 (default values recommended by the CIE for reference conditions: kl = kc = kh = 1). These values are recommended by the original developers of the CIEDE2000 formula for reference conditions and are accepted as standard parametric factors in non-textile applications, including dentistry.

The obtained ΔE₀₀ values were interpreted according to the perceptibility threshold (PT = 0.8 ΔE₀₀) and acceptability threshold (AT = 1.8 ΔE₀₀) established in the dental literature [38] (Table 1).

**Table 1:** Dental CIEDE2000 (ΔE₀₀) Clinical threshold values

ΔE₀₀ Range	Clinical Interpretation	Criterion
< 0.8	Clinically imperceptible	50:50% PT
0.8–1.8	Perceptible but acceptable	PT–AT range
> 1.8	Clinically unacceptable	50:50% AT

*PT *Perceptibility Threshold, *AT *Acceptability Threshold. Threshold values were obtained from a multicenter study with 175 observers

#### Metamerism index calculation

To directly quantify the metameric behavior of each specimen, a metamerism index (MI) was calculated based on the principles defined in the ISO 18314-4:2020 standard and adapted to the CIEDE2000 color difference framework [39]:

$$\mathrm{MI}(\mathrm D65\rightarrow\mathrm{test})\;=\;\vert\triangle{\mathrm E}_{\circ\circ}(\mathrm{test}\;\mathrm{light}\;\mathrm{source})\;-\;\triangle{\mathrm E}_{\circ\circ}(\mathrm D65)\vert$$

Three separate MI values were calculated for each specimen: MI(D65→D50), MI(D65→Incandescent), and MI(D65→F11). The overall metamerism index (MI_overall) was obtained by calculating the arithmetic mean of these three values.

MI values approaching zero indicate that color matching remained stable regardless of light source variation (minimal metameric effect), whereas higher MI values indicate more pronounced light source-dependent color shift. MI values were interpreted using thresholds accepted in the dental literature [39]: MI < 0.8 (clinically imperceptible metameric effect), MI 0.8–1.8 (perceptible but acceptable), MI > 1.8 (clinically unacceptable metameric effect).

All calculations were performed using Konica Minolta SpectraMagic NX software (v2.33, Osaka, Japan), and data were exported to IBM SPSS Statistics 27.0 (IBM Corp., Armonk, NY, USA) for statistical analysis.

### Statistical analysis

All ΔE₀₀ color difference data were analyzed using IBM SPSS Statistics v27.0 (IBM Corp., Armonk, NY, USA). The normality assumption was assessed using the Shapiro–Wilk test, and homogeneity of variance was evaluated using the Levene test. Data showed a normal distribution (*p* > 0.05). Although homogeneity of variance was violated in some groups (*p* < 0.05), parametric analysis was deemed appropriate owing to the robustness of mixed ANOVA against this violation.

Main effects and interactions were assessed using mixed-design repeated-measures ANOVA (within-subjects factor: light source; between-subjects factors: anodization color and composite type). Post hoc comparisons for significant differences were performed using Bonferroni correction. MI values were analyzed using two-way ANOVA, and significant differences were examined using the Tukey HSD post hoc test. The level of statistical significance was set at *p* < 0.05 for all analyses.

## Results

All ΔE₀₀ color difference values were calculated relative to the nonanodized (gray, 0 V) titanium + composite reference group. This reference group was defined as ΔE₀₀ = 0 under the D65 light source.

### Mixed-Design repeated-measures ANOVA

The Shapiro–Wilk test confirmed that the data were normally distributed (*p* > 0.05). Mixed-design repeated-measures ANOVA revealed statistically significant main effects and interactions (Table 2):

**Table 2:** Mixed-design repeated-measures ANOVA results

Source	F	Df	*p*	η²
Between-subjects effects				
Anodization color	696.276	1, 44	< 0.001	0.941
Composite type	881.074	1, 44	< 0.001	0.952
Anodization × Composite	29.554	1, 44	< 0.001	0.402
Within-subjects effects				
Light source	47.921	1.586ᵃ, 69.782ᵃ	< 0.001	0.521
Light × Anodization	9.929	1.586ᵃ, 69.782ᵃ	< 0.001	0.184
Light × Composite	40.108	1.586ᵃ, 69.782ᵃ	< 0.001	0.477
Light × Anodization × Composite	9.865	1.586ᵃ, 69.782ᵃ	< 0.001	0.183

ᵃ Degrees of freedom (df) for within-subjects effects reflect the Greenhouse–Geisser correction

### Light source main effect and interactions

The light source factor showed a statistically significant main effect on ΔE₀₀ values (F(1.586, 69.782) = 47.921; *p* < 0.001; η² = 0.521). Bonferroni-corrected pairwise comparisons (Table 3) revealed distinct color difference patterns among light sources.

**Table 3:** Bonferroni-corrected pairwise comparisons of light sources

Comparison	Mean Difference (ΔE₀₀)	*p*-value
D65 vs. D50	−0.086	< 0.001*
D65 vs. Incandescent (A)	−0.101	< 0.001*
D65 vs. F11	0.006	1.000
D50 vs. Incandescent (A)	−0.015	1.000
D50 vs. F11	0.092	< 0.001*
Incandescent (A) vs. F11	0.107	< 0.001*

*Significant at *p* < 0.05 after Bonferroni correction (adjusted α = 0.0083; for 6 comparisons)

Mean ΔE₀₀ values across light sources were as follows: D65 (2.201 ± 0.026), D50 (2.288 ± 0.026), F11 (2.196 ± 0.025), and Incandescent A (2.302 ± 0.029). No clinically significant difference was found between D65 and F11 (*p* = 1.000; mean difference = 0.006 ΔE₀₀), whereas all other comparisons were statistically significant (*p* < 0.001). The greatest color shift was observed under the Incandescent A light source.

The interaction between light source and anodization color was statistically significant (F(1.586, 69.782) = 9.929; *p* < 0.001; η² = 0.184). Similarly, the interaction between light source and composite type was also significant (F(1.586, 69.782) = 40.108; *p* < 0.001; η² = 0.477). The three-way interaction (Light × Anodization × Composite) also reached statistical significance (F(1.586, 69.782) = 9.865; *p* < 0.001; η² = 0.183). These findings indicate that the effect of light source on color matching varies depending on both anodization color and composite type.

### Anodization color and composite type combinations

The four anodization–composite combinations exhibited distinct color difference patterns across all light sources (Table 4). The yellow anodization with 3 M composite combination (Yellow-3 M) showed the lowest ΔE₀₀ values, whereas the pink anodization with Tokuyama composite combination (Pink-Tokuyama) exhibited the highest color differences.

**Table 4:** Mean ± SD ΔE₀₀ values under different light sources

Group	D65	D50	Incandescent (A)	F11
Yellow-3Mᵃ	0.94 ± 0.11	0.97 ± 0.10	0.97 ± 0.09	0.91 ± 0.11
Yellow-Tokuyamaᵇ	2.07 ± 0.17	2.10 ± 0.17	2.27 ± 0.19	1.99 ± 0.14
Pink-3Mᶜ	2.61 ± 0.16	2.70 ± 0.17	2.72 ± 0.20	2.63 ± 0.16
Pink-Tokuyamaᵈ	3.68 ± 0.27	3.89 ± 0.27	3.97 ± 0.31	3.76 ± 0.25

Different superscript letters (^a, b, c, d^) indicate statistically significant differences between groups (*p* < 0.05). Values were calculated from *n* = 12 specimens per group

Yellow-3 M combination demonstrated color differences close to the perceptibility threshold (0.91–0.97 ΔE₀₀), remaining within the clinically acceptable range. The Yellow-Tokuyama combination exhibited values within the perceptible but acceptable range (0.8 < ΔE₀₀ < 1.8) under D65, D50, and F11, but exceeded the perceptibility threshold under Incandescent A (ΔE₀₀ = 2.27). The Pink-3 M and Pink-Tokuyama combinations showed clinically unacceptable color differences exceeding the acceptability threshold (AT = 1.8) under all light sources.

### Anodization color main effect

The main effect of anodization color was statistically highly significant (F(1, 44) = 696.276; *p* < 0.001; η² = 0.941). Pink anodized titanium (mean ΔE₀₀ = 2.925 ± 0.036) showed significantly higher color differences than yellow anodized titanium (mean ΔE₀₀ = 1.569 ± 0.036) under all conditions. The mean difference between yellow and pink anodization was 1.356 ΔE₀₀ units (95% CI: 1.252–1.460; *p* < 0.001), corresponding to a clinically distinct and unacceptable level of difference.

### Composite type main effect

The main effect of composite type was also statistically highly significant (F(1, 44) = 881.074; *p* < 0.001; η² = 0.952). Tokuyama composite (mean ΔE₀₀ = 3.010 ± 0.036) exhibited significantly higher color differences than 3 M composite (mean ΔE₀₀ = 1.484 ± 0.036) under all conditions. The mean difference between 3 M and Tokuyama composites was 1.525 ΔE₀₀ units (95% CI: 1.422–1.629; *p* < 0.001), indicating a clinically distinct and unacceptable level of difference.

### Anodization color × composite type interaction

**Fig. 4:**
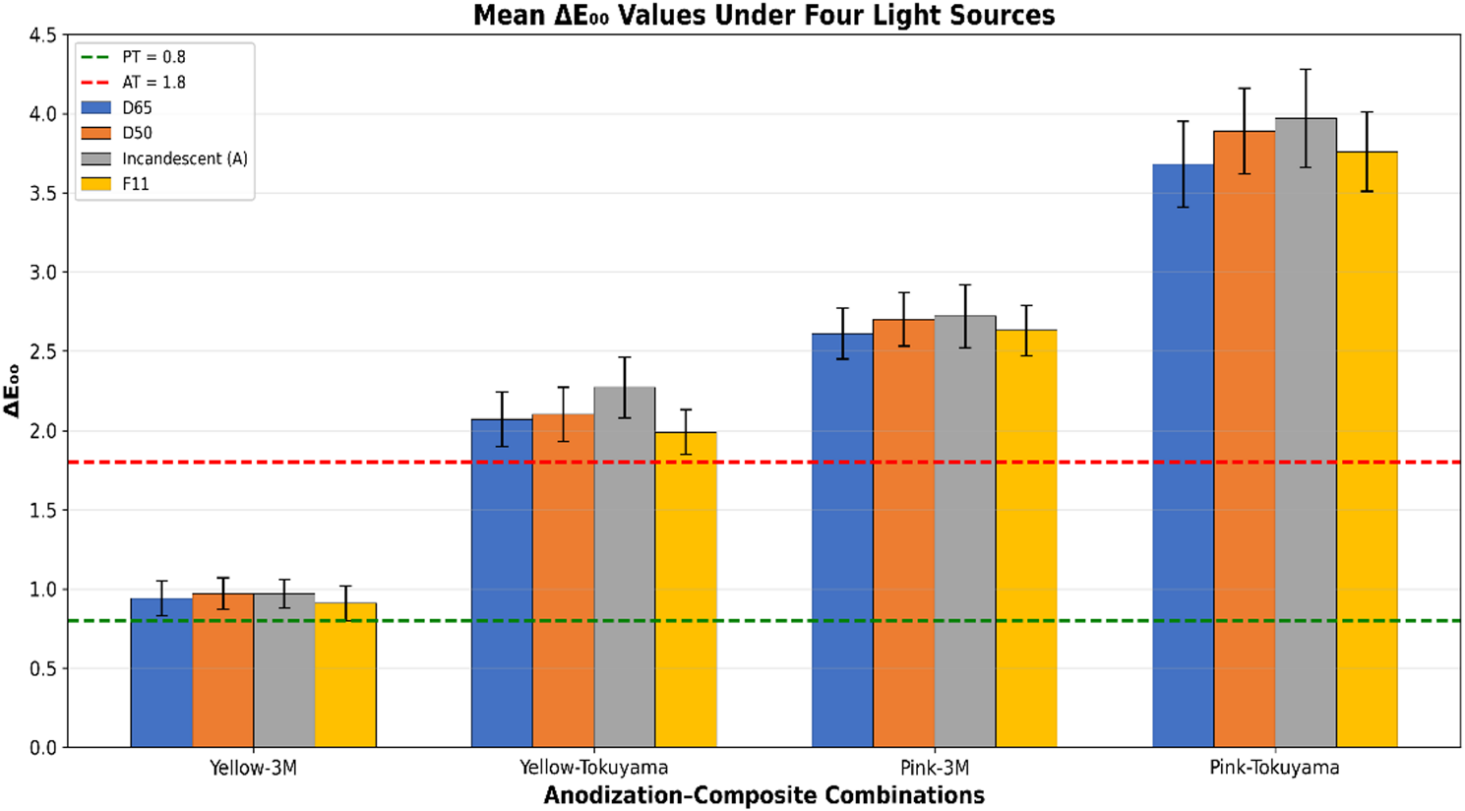
Mean CIEDE2000 color differences (ΔE₀₀) for experimental groups across four standard illuminants. Error bars represent standard deviation (*n* = 12 per group). Statistical comparisons performed using mixed-design repeated-measures ANOVA with Bonferroni post hoc test

The interaction between anodization color and composite type was statistically significant (F(1, 44) = 29.554; *p* < 0.001; η² = 0.402). This interaction indicates that the effect of anodization color varies depending on composite type (Fig. 4). The effect of pink anodization on color difference was more pronounced with Tokuyama composite (ΔE₀₀ = 3.827 ± 0.051) compared with 3 M composite (ΔE₀₀ = 2.192 ± 0.051). In yellow anodization, this difference was less pronounced (3 M: 0.846 ± 0.051; Tokuyama: 2.022 ± 0.051).

### Metamerism index analysis

Metamerism index (MI) values were calculated for each specimen using the formula: MI = |ΔE₀₀(test light source) − ΔE₀₀(D65)|. Three MI values were obtained for each specimen (MI_D50, MI_Incandescent, MI_F11), and the overall MI (MI_overall) was calculated as the arithmetic mean of these three values (Table 5).

**Table 5:** Metamerism index (MI) values for experimental groups

Group	MI_D50	MI_Incandescent	MI_F11	MI_overall	Interpretation
Yellow-3Mᵃ	0.032 ± 0.016	0.042 ± 0.021	0.030 ± 0.032	0.034 ± 0.018	Imperceptibleᵈ
Yellow-Tok.ᵇ	0.078 ± 0.045	0.161 ± 0.095	0.073 ± 0.045	0.104 ± 0.058	Imperceptibleᵈ
Pink-3Mᵇ	0.043 ± 0.035	0.097 ± 0.080	0.149 ± 0.065	0.096 ± 0.037	Imperceptibleᵈ
Pink-Tok.ᶜ	0.211 ± 0.062	0.297 ± 0.166	0.105 ± 0.051	0.204 ± 0.082	Imperceptibleᵈ
Values are presented as mean ± standard deviation (*n* = 12/group)					

Different superscript letters (^a, b, c^) indicate statistically significant differences in MI_overall according to Tukey HSD post hoc test (*p* < 0.05). Groups sharing the same letter (Yellow-Tokuyama and Pink-3 M, both “b”) are statistically equivalent (*p* = 1.000). ᵈMI < 0.8: clinically imperceptible metameric effect (Paravina et al., 2015)

Two-way ANOVA revealed statistically significant main effects on MI values for both anodization color (F(1, 44) = 26.699; *p* < 0.001; η² = 0.378) and composite type (F(1, 44) = 31.865; *p* < 0.001; η² = 0.420). Pink anodization groups showed higher MI values compared with yellow anodization groups (mean = 0.150 ± 0.083 vs. 0.069 ± 0.055). Similarly, Tokuyama composite groups exhibited higher MI values than 3 M composite groups (mean = 0.154 ± 0.087 vs. 0.065 ± 0.042). The interaction between anodization and composite was not statistically significant (F(1, 44) = 1.534; *p* = 0.222), indicating that the effects of these factors on MI are independent and additive.

Tukey HSD post hoc test identified three homogeneous subgroups (Table 6): (1) Yellow-3 M with the lowest MI value (0.034 ± 0.018), significantly different from all other groups; (2) Yellow-Tokuyama and Pink-3 M showing statistically equivalent MI values (0.104 ± 0.058 and 0.096 ± 0.037, respectively; *p* = 1.000), despite different material combinations; and (3) Pink-Tokuyama with the highest MI value (0.204 ± 0.082), significantly different from all other groups. This pattern reveals that different material combinations can exhibit similar metameric behavior when their individual effects counterbalance each other.

**Table 6:** Tukey HSD pairwise comparisons

(I) Group	(J) Group	Mean Difference (I − J)	*p*-value
Yellow-3 M	Yellow-Tokuyama	−0.069	0.033*
Yellow-3 M	Pink-3 M	−0.062	0.077
Yellow-3 M	Pink-Tokuyama	−0.170	< 0.001*
Yellow-Tokuyama	Pink-3 M	0.008	1.000
Yellow-Tokuyama	Pink-Tokuyama	−0.101	0.001*
Pink-3 M	Pink-Tokuyama	−0.108	< 0.001*

*Significant difference at *p* < 0.05. No significant difference was found between Yellow-Tokuyama and Pink-3 M (*p* = 1.000)

**Fig. 5:**
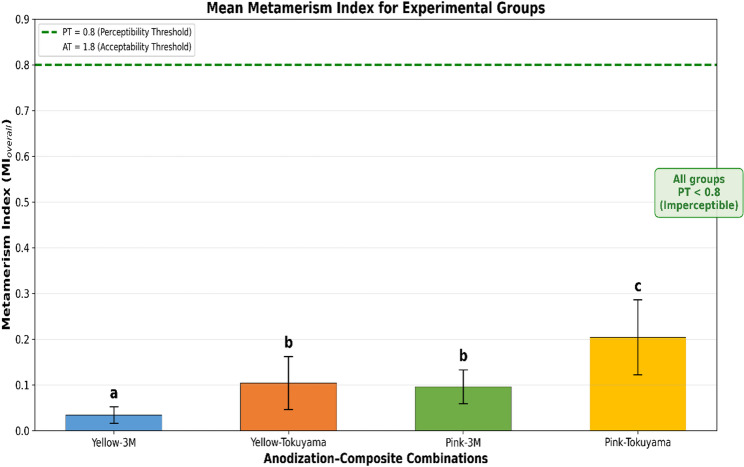
Mean metamerism index (MI) values for experimental groups. Error bars represent standard deviation (*n* = 12 per group). MI quantifies color stability across different lighting conditions. Statistical comparisons performed using two-way ANOVA with Tukey HSD post hoc test

Critically, all observed MI values remained well below the perceptibility threshold (PT = 0.8); even the highest value (Pink-Tokuyama: 0.204 ± 0.082) represented only 26% of this threshold. The maximum individual MI value recorded across all 48 specimens was 0.350, which is still less than half of the PT. This demonstrates clinically imperceptible metameric effects under all experimental conditions, despite statistically significant differences among groups (Fig. 5). The statistical differences in MI values, although significant, do not translate into clinical relevance, as all combinations provide color stability below the human visual perception threshold.

## Discussion

This study evaluated the color matching and metameric behavior of two composite resins applied onto yellow and pink anodized Grade 5 titanium surfaces under four standard illuminants. The findings demonstrated that anodization color (η² = 0.941), composite type (η² = 0.952), and light source (η² = 0.521) significantly influenced ΔE₀₀ values, leading to the rejection of H₀₁, H₀₂, and H₀₃ (all *p* < 0.001). The metamerism index remained below the perceptibility threshold (MI < 0.8) in all experimental groups, confirming H₀₄. These results indicate that while material selection critically determines color matching performance, anodized titanium–composite systems exhibit stable color appearance across different lighting conditions.

### Material effects on color matching

Anodization color and composite type were the two dominant factors influencing ΔE₀₀ values, with effect sizes exceeding 0.9 for both variables. Pink anodization produced significantly higher color differences than yellow anodization (2.925 vs. 1.569 ΔE₀₀; *p* < 0.001), while Tokuyama Estelite Sigma Quick exhibited higher color differences than 3 M Filtek Z250 (3.010 vs. 1.484 ΔE₀₀; *p* < 0.001).

The superior color matching of yellow anodization can be attributed to the optical properties of the titanium oxide layer. Different anodization colors result from variations in oxide layer thickness; yellow anodization is associated with an oxide layer of approximately 50–60 nm, whereas pink anodization produces a thickness of approximately 100–120 nm [40]. Thicker oxide layers exhibit different light absorption and reflection properties at various wavelengths based on the principle of interference optics [41]. The thicker oxide structure of pink anodization appears to have a lower capacity to mask the composite resin color compared with yellow anodization, resulting in greater color discrepancy from the gray titanium reference. In clinical practice, yellow anodization is used to mimic dentin color, whereas pink anodization is preferred for achieving esthetic harmony with peri-implant soft tissues [8]. Our findings suggest that yellow anodization not only serves its intended tissue-matching purpose but also provides better optical compatibility with overlying composite resins.

The difference between composite types is related to filler particle characteristics and their influence on optical behavior. Filler particle size, distribution, and content significantly affect light scattering and transmission within composite resins [42, 43]. The supra-nano spherical filler system of Tokuyama Estelite (mean particle size 0.2 μm) exhibited lower substrate masking capacity compared with the microhybrid filler system of 3 M Filtek Z250 (mean particle size 0.6 μm). This finding is consistent with Yamaguchi et al. [22], who reported that supra-nano filler composites produce structural color effects that may interact with the underlying substrate color. Additionally, differences in refractive index compatibility between matrix and filler components may influence translucency and consequently color matching performance [44].

The significant interaction between anodization color and composite type (F = 29.554; *p* < 0.001; η² = 0.402) demonstrated that material effects are not independent. The impact of pink anodization on color difference was more pronounced with Tokuyama composite (ΔE₀₀ = 3.827) than with 3 M composite (ΔE₀₀ = 2.192), whereas in yellow anodization this difference was smaller (Tokuyama: 2.022 vs. 3 M: 0.846). This interaction indicates that composite selection becomes particularly critical when pink anodization is used, as the Tokuyama composite amplified the color difference produced by the thicker oxide layer.

When interpreted against established clinical thresholds (PT = 0.8; AT = 1.8) [38], the yellow anodization with 3 M Filtek Z250 combination yielded ΔE₀₀ values below the perceptibility threshold under all illuminants (0.91–0.97), representing clinically imperceptible color differences. The yellow anodization with Tokuyama combination fell within the perceptible but clinically acceptable range under most illuminants. In contrast, both pink anodization combinations exceeded the acceptability threshold, indicating clinically unacceptable color differences regardless of composite type. These findings suggest that for esthetically demanding applications such as screw access hole closure in anterior regions and composite veneering in hybrid prostheses, yellow anodization combined with a microhybrid composite provides the most predictable color outcome.

### Light source effect and metamerism

The light source factor showed a statistically significant main effect on ΔE₀₀ values (F(1.586, 69.782) = 47.921; *p* < 0.001; η² = 0.521); however, the mean differences between illuminants were clinically negligible (< 0.11 ΔE₀₀). The highest color differences were observed under Incandescent A illumination (mean 2.302 ΔE₀₀), followed by D50 (2.288 ΔE₀₀), D65 (2.201 ΔE₀₀), and F11 (2.196 ΔE₀₀). The similarity between D65 and F11 results (*p* = 1.000; mean difference = 0.006 ΔE₀₀) suggests that fluorescent illumination commonly used in clinical and office environments produces comparable color evaluation results to standard daylight.

The slightly higher ΔE₀₀ values under Incandescent A can be explained by its spectral power distribution, which has higher emission in the red and yellow wavelengths compared with daylight simulators [45]. This spectral characteristic may differentially affect the perceived color of anodized titanium oxide layers, particularly pink anodization, which has higher spectral reflectance in longer wavelengths. The significant light source × composite interaction (F = 40.108; *p* < 0.001; η² = 0.477) confirmed that different composite resins respond differently to changes in illumination, likely due to their distinct filler-dependent light scattering properties [42, 46, 47].

Despite these statistically significant light-dependent variations, the metamerism index analysis revealed that all titanium–composite combinations exhibited clinically imperceptible metameric behavior. All MI values remained well below the perceptibility threshold (PT = 0.8) [39, 48]. The highest mean MI was observed in the Pink-Tokuyama group (0.204 ± 0.082), representing only 26% of the PT, while the lowest was found in the Yellow-3 M group (0.034 ± 0.018). Even the highest individual MI value recorded across all 48 specimens (0.350) reached only 44% of the PT.

Two-way ANOVA on MI values revealed significant main effects for both anodization color (η² = 0.378) and composite type (η² = 0.420), with no significant interaction (*p* = 0.222), indicating that these factors independently and additively influence metameric behavior. The Tukey HSD analysis identified an interesting pattern: Yellow-Tokuyama and Pink-3 M groups exhibited statistically equivalent MI values (0.104 vs. 0.096; *p* = 1.000), suggesting that different material combinations can produce similar metameric outcomes when their individual optical effects counterbalance each other.

These findings carry important clinical implications. Although spectrophotometry provides objective and reproducible quantification of color differences, clinical color matching is frequently performed using visual or photographic protocols, where variability dependent on observer experience, ambient lighting, and viewing conditions has been reported [49]. The minimal metameric behavior observed in this study suggests that anodized titanium–composite restorations are unlikely to exhibit noticeable color shifts across the lighting environments patients encounter in daily life, including daylight (D65), home incandescent lighting (Illuminant A), and office or clinical fluorescent lighting (F11). This finding is consistent with Corcodel et al. [50], who reported that metameric effects between natural teeth and shade guides remain within clinically acceptable limits under standard illuminants. This color consistency across illuminants represents a favorable characteristic for clinical application.

### Interactions and clinical implications

The significant three-way interaction (Light × Anodization × Composite; F = 9.865; *p* < 0.001; η² = 0.183) confirmed that the effect of each factor varies depending on the levels of the other two factors. This finding underscores the advantage of factorial designs over single-factor studies, as the latter cannot capture these complex interdependencies [51, 52]. Among the two-way interactions, the light source × composite interaction showed the strongest effect (η² = 0.477), indicating that different composite resins respond differently to illuminant changes. The anodization × composite interaction (η² = 0.402) further demonstrated that titanium surface color should not be evaluated independently of composite resin selection. Collectively, these interactions highlight that optimizing a single factor in isolation is insufficient for achieving predictable color outcomes in titanium–composite systems.

The clinical relevance of these findings extends to three principal applications in implant prosthodontics. First, in implant-supported hybrid prostheses, composite resin teeth and artificial gingiva are applied onto a CAD/CAM-milled titanium framework [53]. Modern CAD/CAM-fabricated titanium frameworks offer superior fit, precision, and biocompatibility compared with conventional casting methods [54], and recent systematic reviews have confirmed the long-term clinical success of these frameworks [55]. The color compatibility between the framework and the overlying composite is a determining factor in esthetic success [56]. Our results demonstrated that yellow anodization combined with 3 M Filtek Z250 (ΔE₀₀ = 0.91–0.97) provides clinically imperceptible color differences, making this combination particularly suitable for hybrid prostheses in the anterior region.

Second, in screw-retained implant restorations, screw access holes are sealed with composite resin for both functional and esthetic reasons [14]. The depth of these channels typically ranges between 2 and 3 mm [33], and the gray titanium color beneath translucent composite resins may produce esthetically unacceptable outcomes [57]. Composite thickness and opaquer use have been shown to significantly affect the final color in screw access holes [58]. Kurt et al. [59] reported that a 2 mm composite thickness can be effective in masking the titanium background color; however, opaquer use significantly improved esthetic results. The present study extends these findings by demonstrating that anodization of the titanium surface prior to composite application can substantially improve color matching without requiring additional opaquers, provided that yellow anodization is selected.

Third, chip fractures occurring in monolithic zirconia or polymer-based CAD/CAM restorations over titanium bars are frequently restored via intraoral or laboratory composite repair [15, 16]. The success of such repairs depends on appropriate surface treatment and bonding strategies [16], and the color outcome is directly influenced by the underlying titanium surface. Our findings suggest that pre-anodizing titanium frameworks to yellow before prosthesis fabrication may reduce the risk of color mismatch in cases requiring future composite repair.

The wide range of ΔE₀₀ values observed across material combinations (0.91–3.97) provides an evidence-based framework for clinical decision-making. For esthetically demanding anterior applications, yellow anodization with microhybrid composite is recommended. In posterior regions where esthetic requirements are less critical, other combinations including pink anodization may be acceptable. However, regardless of the clinical scenario, a combined evaluation of anodization color and composite type is essential for achieving optimal esthetic outcomes in implant-supported restorations.

### Limitations and future directions

The present study has several limitations that should be considered when interpreting the findings. First, the in vitro design cannot fully simulate oral cavity dynamics. Aging protocols such as thermal cycling (5–55 °C), mechanical loading, and exposure to staining agents (coffee, tea, red wine) were not applied, which limits the assessment of long-term color stability. Future studies incorporating these aging protocols are recommended to evaluate the clinical durability of color matching in anodized titanium–composite systems.

Second, only two anodization colors (yellow and pink) and two composite resin types were evaluated, with a single composite thickness of 2 mm. Although this thickness represents the clinical standard for posterior restorations and screw access hole closure [31, 33], thinner composite layers may exhibit greater substrate influence on the final color outcome. Investigating additional anodization colors (purple, blue, green, gold), a broader range of composite brands, and multiple composite thicknesses (1, 1.5, 2.5, and 3 mm) would enhance the generalizability of the findings and provide a more comprehensive database for clinical decision-making.

Third, the gray titanium reference used for ΔE₀₀ calculations provides a standardized baseline for isolating the optical effect of anodization; however, it does not represent clinical color targets such as natural tooth or gingival shades. Future studies comparing anodized titanium–composite combinations against tooth shade guides or gingival color standards would strengthen the translational relevance of the findings.

Fourth, color evaluation was performed only objectively using spectrophotometry. Observer-based subjective evaluation from both patient and clinician perspectives was not included. In clinical practice, perceived esthetic acceptability may differ from instrumental measurements. Combining spectrophotometric data with visual assessment protocols under different lighting conditions would provide a more comprehensive understanding of clinical acceptability.

Finally, surface roughness measurements were not performed after polishing. Given that surface texture and gloss can influence spectrophotometric readings, quantifying post-polishing roughness (Ra) values would strengthen the reliability of color measurements in future investigations. Similarly, direct measurement of oxide layer thickness and spectral reflectance properties of anodized surfaces would allow more definitive mechanistic conclusions regarding the relationship between oxide characteristics and color performance.

## Conclusions

This study systematically evaluated the effect of anodization color and composite resin type selection on color matching in three clinical applications: anodized titanium–composite resin hybrid prostheses, composite closure of screw access holes in screw-retained prostheses, and composite repair applications on anodized titanium surfaces. The findings demonstrated that both factors play an important role in esthetic outcomes. Metameric evaluation under different lighting conditions revealed that color stability was at an adequate level for clinical use.

In terms of material selection, the yellow anodization and 3 M Filtek Z250 combination was identified as a suitable option for esthetically compatible outcomes in the closure of screw access holes and composite repair applications in anterior regions. This finding may serve as a guide for clinician preferences in implant-supported restorations where esthetic expectations are high. For achieving optimal esthetic outcomes in screw-retained hybrid prostheses and composite applications on anodized titanium, a combined evaluation of titanium anodization color and composite resin type is recommended.

Clinical trial number: Not applicable. This study does not involve human participants, human data, or clinical intervention.

## Data Availability

The datasets generated and analyzed during the current study are available from the corresponding author upon reasonable request.

## Abbreviations

ANOVA: Analysis of variance
AT: Acceptability threshold
ASTM: American Society for Testing and Materials
CAD/CAM: Computer-aided design / computer-aided manufacturing
CI: Confidence interval
CIE: Commission Internationale de l’Éclairage (International Commission on Illumination)
CIEDE2000: CIE color difference formula, 2000 revision
CNC: Computer numerical control
DC: Direct current
df: Degrees of freedom
F: F-statistic (ANOVA)
F11: CIE standard illuminant F11 (fluorescent, TL84)
ISO: International Organization for Standardization
LED: Light-emitting diode
MAV: Medium area view
MI: Metamerism index
PT: Perceptibility threshold
RM-ANOVA: Repeated-measures analysis of variance
SCI: Specular component included
SD: Standard deviation
Ti-6Al-4V: Titanium–6 aluminum–4 vanadium (Grade 5 titanium alloy)
ΔE₀₀: CIEDE2000 color difference
α: Significance level (alpha)
η²: Partial eta-squared (effect size)
